# Construction Notes

**Published:** 1896-06-13

**Authors:** 


### CONSTRUCTION NOTES.

**New Wing to Hackney Union Infirmary.**

The accommodation here is for 220 patients, which is provided on the ground and on the upper floors, the basement being occupied with the heating chamber, store-room, &c. From each ward a bath-room and lavatory leads off. The arrangement for this extension has been to disconnect all the floors as much as possible; and the staircase is in a wing building connected with the main building only by bridges, the lift for patients and other purposes also being outside. A verandah extends the length of each large ward, with which a fire-escape staircase is connected. All wards and staircases are fire-proof, and the wards will be heated by hot-water pipes and central stoves with descending flues. The ventilation is to be carefully and effectually carried out, and Boyles' ventilators are to be added to the roof. The architect is Mr. W. A. Finch, of Finsbury Pavement. Mr. S. R. Lamble is the builder.

